# PCSK9 inhibitors in real life—Cardiometabolic risk management in dyslipidemic patients in Vienna

**DOI:** 10.1007/s00508-024-02402-9

**Published:** 2024-08-13

**Authors:** M Ferch, C Sert, P Fellinger, A Kautzky-Willer, Y Winhofer-Stöckl

**Affiliations:** Department for Medicine III, Division of Endocrinology and Metabolism, Medical University of Vienna, Währinger Gürtel 18–20, 1090 Vienna, Austria

**Keywords:** Austria, Follow-up, Real-world data, Target-Attainment, Lipoprotein(a)

## Abstract

**Background:**

Proprotein convertase subtilisin kexin type 9 (PCSK9) inhibitors have emerged as important therapeutic options for patients unable to achieve the low-density lipoprotein cholesterol (LDL‑C) target or to tolerate alternative lipid-lowering agents. The aim of this study was to investigate the efficacy of PCSK9 inhibitor treatment in tertiary routine care, by determining the percentage of patients reaching individual LDL‑C target levels 1 year after treatment initiation.

**Patients and methods:**

Patients routinely started on PCSK9 inhibitors at our lipid clinic between 2017 and 2020 were retrospectively analyzed. Attainment of the LDL‑C target, utilization of follow-ups, cardiovascular events and effects on laboratory parameters were investigated.

**Results:**

In this study 347 patients were included, with the majority managed in secondary prevention (94.5%). The LDL‑C target was achieved by 44.9% after ca. 14 months, with differences between statin users and non-users (51.0% vs. 22.7%; *p* < 0.001). The median LDL‑C decreased from 126.00 mg/dL at baseline to 48 mg/dL (−61.6%; −77.00 mg/dL; *p* < 0.001) after ~2 months and to 60 mg/dL (−52.9%; −59.00 mg/dL; *p* < 0.001) after ~14 months. Median lipoprotein(a) levels decreased significantly from 184.0 nmol/L to 165.5 nmol/L (−25.9%; −25.5 nmol/L; *p* = 0.001) after ~2 months, whereas no effects on creatine kinase, amylase and lipase were detectable. Of the patients 15% utilized 4 follow-ups. The PCSK9 inhibitor intolerance occurred in 3.5% of patients.

**Conclusion:**

With the effect of LDL-lowering remaining constant over 14 months, PCSK9 inhibitor treatment showed effective and sustainable LDL‑C lowering in a majority of patients in secondary prevention, bringing them closer to the recommended LDL‑C goal, particularly those under concomitant statin medication. Treatment with PCSK9 inhibitors appears to be well-tolerated, confirming data from clinical trials in real life.

**Supplementary Information:**

The online version of this article (10.1007/s00508-024-02402-9) contains supplementary material, which is available to authorized users.

## Introduction

Proprotein convertase subtilisin kexin type 9 inhibitors (PCSK9i) are an approved class of antilipemic agents that have emerged as therapeutic options for patients unable to achieve the low-density lipoprotein (LDL-C) target or to tolerate alternative lipid-lowering agents [1]. Imaging studies such as GLAGOV and PACMAN-AMI demonstrated enhanced plaque volume regression and plaque stabilization under PCSK9i-mediated LDL‑C control [2–5], warranting increased lipid lowering. Due to efficient lipid lowering via PCSK9i, a larger proportion of patients are now expected to reach recently lowered LDL‑C targets as recommended for cardiovascular disease (CVD) prevention by the ESC 2019 guidelines [6].

However, LDL‑C target attainment in secondary prevention has previously been shown to be difficult to achieve in national and international clinical practice. In the DA VINCI and the more recent SANTORINI study and their respective subgroup analyses for Austria [7–10], the majority of patients failed to attain the respective LDL‑C goals, which is in part due to low coverage of potent LDL‑C lowering treatment including PCSK9is, demonstrating substantial potential for improvements in lipid care for Austrian patients. Therefore, the aim of the study was to investigate the efficacy of PCSK9i treatment in routine care in a tertiary care lipid clinic and to examine whether a comprehensive use of PCSK9i brings patients of this vulnerable cohort closer to the individual LDL‑C targets.

## Methods

In this single-center, observational study, a retrospective analysis of data from electronic patient charts was performed. All patients who were routinely prescribed with an at the time available PCSK9i (alirocumab 75 mg or 150 mg or evolocumab 140 mg; every other week) between 1 March 2017 and 31 March 2020 at our tertiary out-patient lipid clinic were included. Attainment of LDL‑C target (per ESC/EAS guidelines 2019 [6]) after treatment initiation was the primary objective. Utilization of follow-ups, cardiovascular (CV) events suffered within a minimum observation period of 2 years, differences between target attainers and non-attainers and effects on laboratory parameters including LDL‑C, lipoprotein(a) (lp(a)), creatine kinase (CK), amylase and lipase were secondary objectives. Additional to the in-house electronic chart review, a nationwide electronic death record matching was undertaken.

This study was approved by the Human Ethics Committee of the Medical University of Vienna (Ethics Committee number: 1323/2021)*. *Statistical analyses were performed using IBM Statistical Package for the Social Sciences, Version 27.0 (IBM SPSS Statistics for Macintosh, Armonk, NY: IBM Corp. USA). Continuous data were presented as means and standard deviations or medians and interquartile ranges, however appropriate. Student’s *t* test, χ^2^-test and Mann-Whitney U‑test were used for comparing metric, nominal, and ordinal data, respectively. For comparison of paired parameters, Student’s *t*-test or Wilcoxon signed ranks test for non-parametric data was used, respectively, χ‑test was used for comparing target attainment between patient groups. Graphics were created using SPSS version 27.0. Statistical significance was defined as *p* < 0.05, two-sided.

## Results

### Baseline characteristics

A total of 347 patients were identified who were prescribed a PCSK9i during the 3‑year period. The majority of the patients were male (58.5%) and managed in secondary prevention (94.5%), with a mean with age of 62 years and mean body mass index (BMI) of 28.2 kg/m^2^, see Table 1.

**Table 1:** Characteristics of the total cohort (*n* = 347) at baseline

Characteristics	
*Age (years)*	62.2 (± 11.4)
*Sex (% male)*	58.5
*BMI (kg/m*^*2*^*)*	28.2 (± 4.8)
TC (mg/dL)	205.0 (168.0–252.0)
TG (mg/dL)	149.0 (96.3–204.8)
LDL‑C (mg/dL)	126 (92.6–171.0)
HDL‑C (mg/dL)	49.0 (40.0–60.0)
Non-HDL‑C (mg/dL)	155.0 (117.0–207.5)
Lp(a) (nmol/L)	127.5 (12–243.5)
HbA1c (%)	5.7
Creatine kinase (U/L)	101 (75–217)
CRP (mg/dL)	0.22 (0.09–0.52)
*Familial hypercholesterolemia (%)*	11.8
*Diabetes mellitus, type II (%)*	23.6
*ASCVD (%)*	94.5
*PCSK9i agent (%)*	
Evolocumab	58.2
Alirocumab	41.8
*LDL‑C target (%)*	
<40 mg/dL	10 (2.9)
<55 mg/dL	319 (91.9)
<70 mg/dL	18 (5.2)
*ASCVD*	328 (94.5)
Coronary artery disease (%)	133 (38.3)
Carotid artery stenosis (%)	46 (13.3)
Peripheral artery disease (%)	10 (2.9)
Polyvascular disease (%)	139 (40.0)
*Lipid lowering therapy*	
Statins (%)	52.4
Low intensity (%)	0.3
Moderate intensity (%)	8.6
High intensity (%)	43.5
Ezetimibe (%)	59.7
Nutraceuticals (%)	6.9
Lipoprotein apheresis (%)	1.7
Statin intolerance (%)	62.8
Ezetimibe intolerance (%)	23.9

*BMI* body mass index, *TC* total cholesterol, *TG* triglycerides, *LDL-C* low-density lipoprotein cholesterol, *HDL-C* high-density lipoprotein cholesterol, *Lp(a)* lipoprotein(a), *HbA1c* hemoglobin A1c, *CRP* C-reactive protein, *ASCVD* atherosclerotic cardiovascular disease, *PCSK9i* proprotein convertase subtilisin kexin type 9 inhibitor

Values given as means (±standard deviation), medians (interquartile range) or counts (%)

### Utilization of follow-up examinations

Of the patients 65.9% (*n* = 288), 48.1% (*n* = 167), 26.8% (*n* = 93) and 15.0% (*n* = 52) utilized 1, 2, 3, or 4 follow-up examinations after a median of 2, 9, 14 and 20 months, respectively.

### LDL-C target attainment

**Fig. 1:**
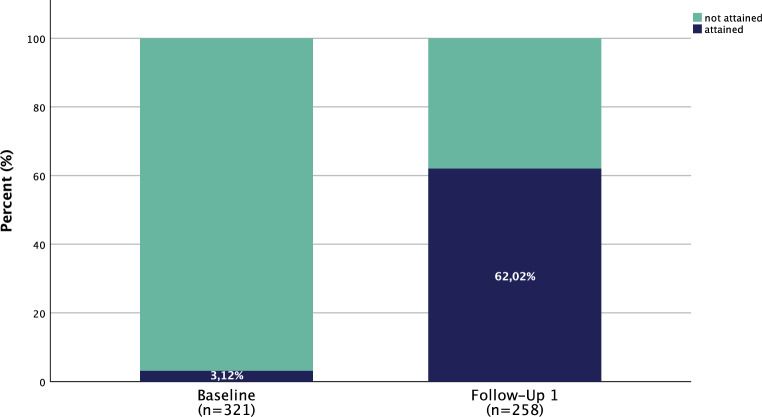
Low-density lipoprotein cholesterol (LDL‑C) target attainment at baseline and first follow-up, *n* indicating the number of patients with LDL‑C values available

Individual LDL‑C target was attained by 62% of patients after a median of 2 months of treatment at the first follow-up as illustrated in Fig. 1. For target attainment at the following visits, see Supplemental Fig. 1.

**Fig. 2:**
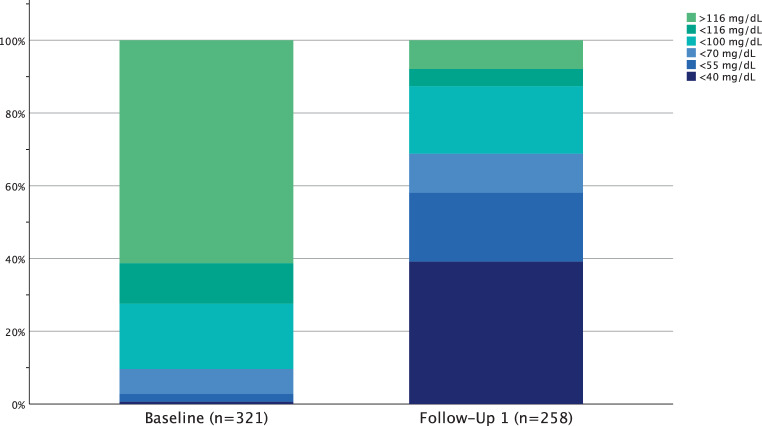
Attained low-density lipoprotein cholesterol (LDL‑C) range at baseline and first follow-up visit, *n* indicating the number of patients with LDL‑C values available

The ranges of attained LDL‑C at baseline and the first follow-ups are illustrated in Fig. 2. Further follow-up visits are illustrated in Supplemental Fig. 2, with a decreasing number of participants observed.

### Attainers vs. non-attainers

There were significant differences between target attainers and non-attainers regarding the prevalence of genetically verified familial hypercholesterolemia (FH, 10.0% vs. 19.4%; *p* = 0.033), statin intolerance and statin use (53.8% vs. 80.6%; *p* < 0.001 or 51.0% vs. 22.7%; *p* < 0.001, respectively), and ezetimibe intolerance and ezetimibe use (20.0% vs. 33.7%; *p* = 0.014, or 58.1% vs. 35.4%; *p* < 0.001, respectively). In total, 54.3% of FH patients, 47.9% of statin-intolerant patients and 50.8% of ezetimibe-intolerant patients did not attain individual LDL‑C target at the first follow-up. Attainment compared to non-attainment did not significantly differ by sex (60.0% vs. 58.2% males; *p* = 0.771), diabetes (28.1% vs. 22.4%; *p* = 0.313), or across individual LDL‑C target levels (in those with an LDL‑C goal of < 55 mg/dL the attainment was 63.0%, in those with <40 mg/dL 44.4%, and 54.5% in those with <70 mg/dL; *p* = 0.462).

### Course of laboratory parameters

**Fig. 3:**
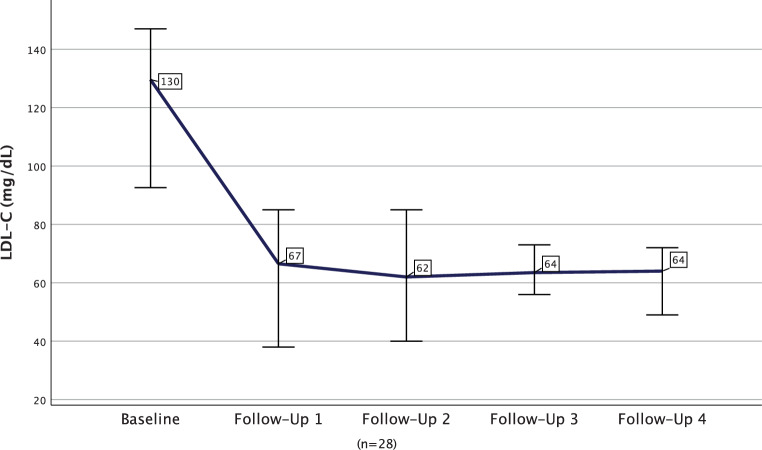
Longitudinal course of median low-density lipoprotein cholesterol (LDL‑C) among participants without missing LDL‑C measurements in any of the visits (*n* = 28)

**Fig. 4:**
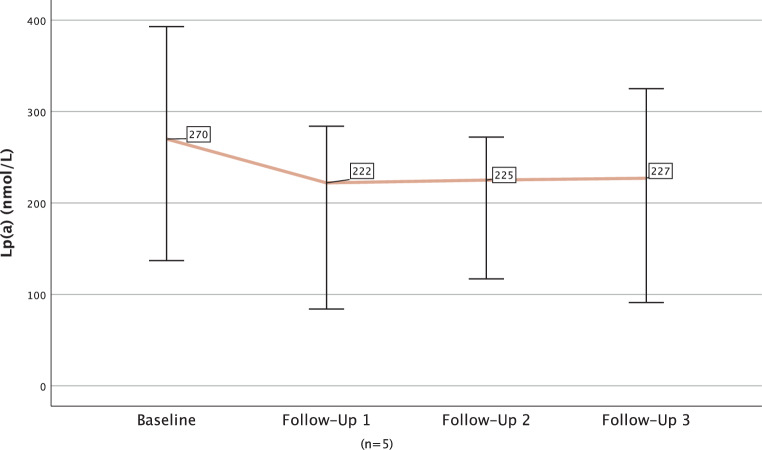
Longitudinal course of median lipoprotein(a) (lp(a)) among patients without missing lp(a) values in any of the visits (*n* = 5)

**Fig. 5:**
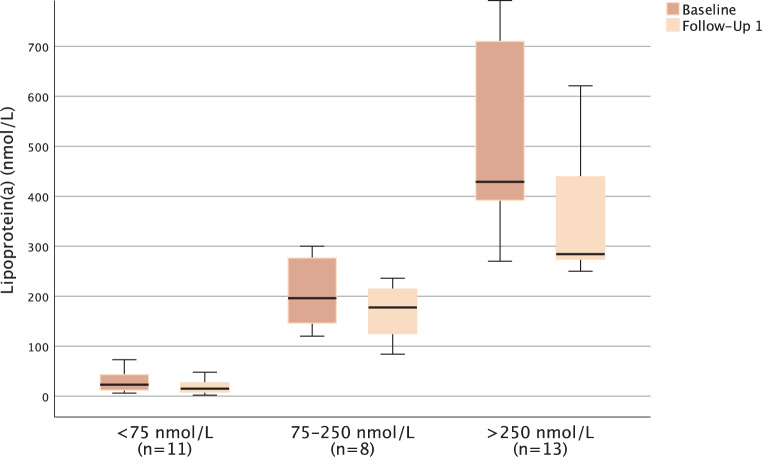
Course of lipoprotein(a) (lp(a)) after initiation of proprotein convertase subtilisin kexine type 9 (PCSK9) inhibitor therapy. Levels of lp(a) at baseline (dark) and follow-up 1 (light) across ranges of normal (< 75 nmol/L) (left), elevated (75–250 nmol/L) (middle) and extremely elevated (> 250 nmol) (right) lp(a)

The median LDL‑C decreased from 126.00 mg/dL at baseline to 48 mg/dL (median difference −61.6%; −77.00 mg/dL; *p* < 0.001) after ~2 months and to 60 mg/dL (median difference −52.9%; −59.00 mg/dL; *p* < 0.001) after ~14 months in the overall cohort. In a longitudinal approach among those without missing values in any of the visits, LDL‑C values can be demonstrated to remain constantly lowered throughout four follow-ups (Fig. 3). Lp(a) levels were available for 22% (*n* = 32) of patients at both baseline and first follow-up and were significantly lowered from a median of 184.0 nmol/L at baseline to a median of 165.5 nmol/L after ~2 months (median difference −25.9%; −25.5 nmol/L; *p* = 0.001; ranging from 62 nmol/L to −440 nmol/L). Throughout the course of three follow-ups, data on lp(a) were available for 5 patients and showed no notable changes after the first follow-up (Fig. 4). Levels of lp(a) decreased across ranges of normal (< 75 nmol/L), elevated (75–250 nmol/L) and extremely elevated (> 250 nmol) lp(a) from baseline to first follow-up (−9 nmol/L; −38.7%, *p* = 0.008; −19.0 nmol/L, −12.68%, *p* = 0.401; or −93.0 nmol/L, −26.5%, *p* = 0.006, respectively) as shown in Fig. 5. No effects on CK, amylase, lipase or C-reactive protein (median differences: −2.5 U/l, *p* = 0.154; 0.0 U/l, *p* = 0.657, +2.0 U/l, *p* = 0.308 or 0.015 mg/dl, *p* = 0.329, respectively) were detectable. Lipase and amylase levels, however, were unavailable in a substantial proportion of patients (available in 35.4%).

### Cardiovascular events and death under treatment

In total, five CV events were recorded during a minimum observation period of 2 years. Additionally, three deaths were attributed to ASCVD out of eight total documented deaths (three of which unspecified, one non-ASCVD and one non-atherosclerotic-cardiac failure).

### Adverse events

Side effects attributable to newly initiated PCSK9i treatment were reported in 5.8% (*n* = 20) of the cases. The symptoms documented included allergic skin reaction, joint pain and vertigo, leading to discontinuation of treatment due to PCSK9i intolerance in 3.5% (*n* = 12) of the cases.

## Discussion

In order to prevent CV events, lower LDL‑C target levels have to be achieved as per 2019 [6], which, in turn leads to increasing difficulties in attaining LDL‑C goals for a significant proportion of patients [7], necessitating more potent lipid lowering. Failure to reach target levels may not only be due to inability to tolerate appropriate statin doses or lack of adherence but specifically concern certain patient groups, such as very high cardiovascular risk and FH patients, requiring additional antilipemic agents [1]. This is confirmed by the fact that patients with FH, statin intolerance and ezetimibe intolerance significantly struggled to reach goals in our cohort, stressing the need for alternative agents for exhaustive combination therapy.

With the recently approved PCSK9is, a larger proportion of patients were expected to reach targets; however, attainment specifically for patients receiving PCSK9is has not been investigated before in Vienna. A study on PCSK9is from other clinics in Austria showed a 50% decrease in LDL‑C levels in a real-life setting with 70.5% of patients achieving ESC/EAS 2016 guideline-recommended LDL‑C levels [11]. A recent study from Graz on patients with PCSK9i therapy reported 61.2% of the patients achieving LDL‑C levels of < 70 mg/dl, and 44.1% achieving LDL‑C levels of < 55 mg/dl [12], whereas in our study target attainment was achieved in 63% of patients with a goal <55 mg/dl after a median of 2 months.

Previous European studies focusing on population-wide numbers in contrast to our tertiary care cohort, report LDL‑C goal attainment rates of 20.1% as the most recent (SANTORINI [9]), 33% (DA VINCI [7]), or 46.9% (EUROASPIRE V [13]) in 2018. Analogously, LDL‑C target level attainment in Austria has been reported in the past. Despite target values being less stringent per former ESC guidelines of 2007 [14], 2011 [15] and 2016 [16], and PCSK9is was not available at that time, the potential for improvements in lipid care for Austrian patients was consistently demonstrated through goal attainment rates of 39.9% [17], 41% [18], 47.7% [19] 37.2% [20] 40.3% [21] per ESC/EAS 2007 guidelines and 13.9% [21] per ESC/EAS 2011 guidelines. A recently published post hoc analysis on 293 Austrian participants recruited in 8 centers of the cross-sectional European DA VINCI study, reported 38% goal attainment as per ESC/EAS 2019 guidelines, indicating a good performance in matters of target attainment in our cohort during the observed period [8]; however, further studies from other centers may be needed for up to date conclusions on the treatment situation in Austrian patient cohorts.

With increasing numbers of follow-ups, the study population under observation and consequently, the proportion of patients attaining the individual LDL‑C goal is however decreasing, attributable to attrition bias, as those patients with difficulties in attainment are likely being followed up more extensively, whereas those without problems in reaching target levels are likely to be exempted from follow-up examinations earlier.

The LDL‑C was significantly and sustainably lowered in the medium term, as demonstrated in the longitudinal observation in those without any missing values. Lp(a) was lowered by a median of 25.9% which is in line with previously reported decreases of up to 25–30% [22]. None of those with elevated lp(a) reached normal levels after treatment, confirming the need for further more effective pharmacotherapy for lp(a) lowering.

No adverse changes in parameters indicative of side effects (lipase, amylase, CRP) were noted in this study, CK improved likely due to the omission of statins at PCSK9i initiation, indicating excellent tolerability, agreeing with previous trials.

As observation is limited to in-house documentation and death registry matching, any (nonfatal) cardiovascular events happening after treatment initiation could not be assessed due to lacking systematic and comprehensive follow-up visits (only 15% long-term data available), as patients treated in other hospitals were not captured.

The utilization of follow-ups might be in part influenced by coronavirus disease 2019 (COVID 19) lockdowns with limited out-patient care in 2020, which affects the minimum observation period of the majority of the patients. First prescription rates however, are representative, as inclusion was limited to the time range from the start of first-prescriptions at our center until the first COVID 19 lockdown (March 2020).

In conclusion, this is the first comprehensive report on LDL‑C target attainment and CV outcomes specific for patients under PCSK9i treatment from Vienna. With the effect of LDL lowering remaining constant throughout 12 months, PCSK9i treatment shows effective and sustainable LDL‑C lowering in a majority of patients in secondary prevention, bringing them closer to thei recommended LDL‑C goal and emphasizing the importance of combination therapy with synthesis inhibitors such as statins. The PCSK9i treatment appears to be well-tolerated, all in all confirming data from clinical trials in real life.

## Supplementary Information

Supplemental Fig. 1: Low-density lipoprotein cholesterol target attainment at baseline and first follow-up. Notably, the study population decreases during the course of time

Supplemental Fig. 2: Attained low-density lipoprotein cholesterol range at baseline and first follow-up visit
